# Establishment of Mammary Gland Model *In Vitro*: Culture and Evaluation of a Yak Mammary Epithelial Cell Line

**DOI:** 10.1371/journal.pone.0113669

**Published:** 2014-12-05

**Authors:** Mei Fu, Yabing Chen, Xianrong Xiong, Daoliang Lan, Jian Li

**Affiliations:** 1 College of Life Science and Technology, Southwest University for Nationalities, Chengdu, China; 2 Institute of Qinghai-Tibetan Plateau, Southwest University for Nationalities, Chengdu, China; Georgetown University, United States of America

## Abstract

This study aimed to establish yak mammary epithelial cells (YMECs) for an *in vitro* model of yak mammary gland biology. The primary culture of YMECs was obtained from mammary gland tissues of lactating yak and then characterized using immunocytochemistry, RT-PCR, and western blot analysis. Whether foreign genes could be transfected into the YMECs were examined by transfecting the EGFP gene into the cells. Finally, the effect of *Staphylococcus aureus* infection on YMECs was determined. The established YMECs retained the mammary epithelial cell characteristics. A spontaneously immortalized yak mammary epithelial cell line was established and could be continuously subcultured for more than 60 passages without senescence. The EGFP gene was successfully transferred into the YMECs, and the transfected cells could be maintained for a long duration in the culture by continuous subculturing. The cells expressed more antimicrobial peptides upon *S.aureus* invasion. Therefore, the established cell line could be considered a model system to understand yak mammary gland biology.

## Introduction

The mechanisms involved in milk protein expression and udder resistance to pathogens that cause infectious agalactia or secretion of abnormal milk have gained increasing attention because of the commercial value of milk. The key issue in mammary gland biological experiments is selecting an appropriate research model [1]. *In vivo* experiments result in systemic effects on animals; thus, maintaining the environment of mammary glands is difficult [2], [3]. A popular approach is to establish a mammary epithelial cell (MEC) line as a convenient research material [4]. The model should mimic the function of the mammary gland to evaluate its physiological, biochemical, and immunological functions [5]. As of this writing, many MEC lines, such as human [6], mouse [7], [8], bovine [1], [3], [9], pig [10]–[12], buffalo [13], [14], sheep [15], [16], and goat [4], [17], [18], have been established. These cell lines aid in elucidating mammary gland biology. However, yak MECs (YMECs) have not been reported.

Yak (*Bos grunniens*), which lives in the Tibetan Plateau, is important in Tibetan life because it provides meat and milk in a place where few other animals can survive [19], [20]. In terms of milk production, the annual output of yak is low at 150–250 kg compared with that of cow at 3,000–8,000 kg. However, yak milk contains higher total solids (protein, fat, and minerals) of 18%–22% compared with 13%–16% in cow milk. Although selective breeding and modern management techniques can increase yak milk production, the increase is actually limited. Hence, investigations on yak mammary gland biology are necessary to improve milk production. The primary cell line of the mammary gland represents an *in vivo* condition, and maintains organ-specific functions and signal transduction pathways. This type of YMEC can also be used to evaluate cell differentiation during lactation, immune response to bacterial infections, and mammary gland bioreactions [21]. Although cattle and yak belong to the *Bovidae* family, species-specific variance exists between these two species [20]. Therefore, using an YMEC line is more appropriate than using cell lines from other species, such as cattle, to elucidate the specificity of the lactation mechanism of yak. In this study, we established and characterized a primary cell culture of YMEC line. The cell line could respond to lactogenic hormonal induction and express milk proteins. Moreover, YMECs could be transferred with the foreign gene EGFP; thus, YMECs may be used as a model for transgene screening system to identify superior transgenes prior to transgenic animal production. Furthermore, the established cell line could be utilized for further studies on the bacterial infection response of the mammary gland.

## Materials and Methods

### Ethics Statement

All experimental procedures were approved by the Animal Care and Use Committee of the Southwest University for Nationalities, Sichuan, China, and performed in accordance with the animal welfare and ethics guidelines.

### Medium for Cell Culture

Basal growth medium was composed of 90% DMEM/F12 (Hyclone, USA) and 10% fetal bovine serum (FBS, Gibco, USA), which was supplemented with 100 IU/mL penicillin and5 µg/mL streptomycin. To promote the synthesis of milk proteins, the induction medium contained 5 µg/mL insulin-transferring-selenium (Sigma, USA), 5 ng/mL epithelial growth factor (Sigma, USA), 1 µg/mL hydrocortisone (Sigma, USA), and 5 µg/mL progesterone (Sigma, USA). Storage media consisted of 60% DMEM/F12, 30% FBS, and 10% DMSO (Sigma, USA).

### Isolation and Culture of YMECs

Mammary tissues were obtained from a four-year-old mid-lactation yak from a local slaughterhouse (Chengdu, China). The collected fresh tissues were placed in sterilized tubes containing ice-cold Dulbecco's PBS (DPBS, Sigma), and immediately transported to the laboratory. The samples were washed with DPBS containing antibiotics for several times and cut into 1 mm^3^ pieces. The tissues were transferred with a pincet onto clean plastic cell culture dishes (Corning, USA). The culture dishes were inverted and incubated at 37°C under 5% CO_2_. After 4 h, 5 mL of basal medium was added into the culture. The basal medium needed to be replaced with fresh medium every 48 h until the cells were distributed across the bottom of the dish. After that, epithelial cells were enriched by selective detachment with trypsinization using 0.25% trypsin (Gibco, USA). After 2–3 min of trypsinization, detached fibroblast cells were removed by washing with DPBS. The epithelial cells attached to the dish surface were allowed to grow by addition of fresh medium. YMECs were continuously purified using the same method. The purified YMECs were seeded at a density of 5×10^5^ cells in culture flasks, and continuously subcultured up to 60 passages. For cryopreservation, 1×10^6^ cells/mL was suspended in freezing medium.

### Growth Characteristics of Epithelial Cells

The *in vitro* growth pattern of YMECs was assessed by observing their doubling time at 10 th (early passage), 50 th (late passage), and 15 th passages (frozen thawed cells). In brief, 2×10^4^ cells/well was seeded in 12-well culture plates (Corning, USA) containing induction media. Cell number and viability were examined each day in triplicate wells using trypan blue exclusion until 7 d post-seeding. The morphology of cultured cells was also determined using an inverted microscope with phase contrast (Olympus, CKX41, Japan). Bacterial contamination of YMECs was assessed by culturing with antibiotic-free medium, whereas mycoplasma contamination was analyzed using a mycoplasma detection kit (ExCell Bio, China).

### Senescence-associated β-galactosidase assay (SA-β-gal)

SA-β-gal assay kit (sigma, USA) was used to assess cellular senescence. Brifely, YMECs at passage 10 and 60, and MCF-7 cells (a human breast cancer cell line, senescent stage-negative control) were washed with DPBS and fixed for 15 min at room temperature with fixation buffer. The cells were then washed with three times for 3 min each with PBS. Subsequently, the cells were incubated for 12 h at 37°C with SA-β-gal stain solution. Photographs of stained cells were acquired using (Olympus, CKX41, Japan).

### RNA Extraction and ReverseTranscription Reaction

Total RNA from mammary tissue, skin fibroblast cells, YMECs cultured with induction media, and YMECs cultured with basal growth medium was isolated using Trizol (Invitrogen, Shanghai, China) according to the manufacturer's instructions. RNA was quantitatively and qualitatively determined using an ultraviolet spectrophotometer at 260 and 280 nm. RNA integrity was determined using electrophoresis on 1.0% agarose gel. Total RNA was reverse transcribed using a RevertAid Premium first-strand cDNA synthesis kit (Fermentas, USA). The generated cDNA was then amplified using gene-specific primers. The primers for β-casein (CSN2), butyrophilin (BT1N1A1), lactoferrin (LTF), β-lactoglobulin (BLG), and GAPDH were designed using Primer 5.0 software (Table1) and synthesized by Invitrogen (Shanghai, China).

**Table 1:** Primers sequences for RT-PCR on identification of YMECs.

Gene	Primers	Melting temperature (°C)	Product size (bp)
CSN2	F:5′-AGGAACAGCAGCAAACAG-3′	56	579
	R:5′-TTTCCAGTCGCAGTCAAT		
BTN1A1	F:5′-TGTGTTGCTGCTGATAGAGTGTTAG-3′	52	305
	R:5′-CCTCCAAGTTCCTTTATGGGATTTC-3′		
LTF	F:5′-GCCTTTGCCTTGGAATGTA-3′	56	290
	R:5′-AAGTACGGGCGAAGGATTC-3′		
BLG	F:5′-CCCTGAGAGTGTATGTGGAG-3′	57	272
	R:5′-AGGACTTTGTTCTCGTTCAAGG-3′		
GAPDH	F:5′- GC TGGTGCTGAGTAGTTGGTG-3′	60	220
	R:5′- TCTTCTGGGTGGCAGTGATGG-3′		

### Immunofluorescence of YMEC markers

The expression of protein markers (cytokeratin 8, cytokeratin 18, and vimentin) in YMECs was determined by immunocytochemical analysis. Cells were seeded in 12-well culture plates at a density of 2×10^4^ cells/well and cultured until they reached confluence. The cells were washed with DPBS, fixed with 4% paraformaldehyde for 30 min, and treated with 1% Triton X-100 for 5 min. The cells were subsequently blocked at 37°C for 1 h in 1% bovine serum albumin (BSA). The slides were incubated overnight with the primary antibodies for cytokeratin 8 (Abcam, USA), cytokeratin 18 (Abcam, USA), and vimentin (Abcam, USA), which were diluted to1∶50, 1∶100, and 1∶200, respectively. The cells were washed three times for 5 min each with DPBS and then incubated with secondary antibodies, namely, FITC-conjugated monoclonal goat anti-mouse IgG (Beyotime, China) and Cy3-conjugated monoclonal goat anti-mouse IgG (Beyotime, China), for 1 h in the dark. Hoechst 33342 was used as a nuclear counterstain. Lastly, the slides were washed three times and visualized with a fluorescent phase-contrast microscope (Olympus, DP 70, Japan).

### Chromosomal Analysis

Chromosomes were analyzed in exponentially growing cells obtained from three periods (primary, purified, and resuscitated cells). Karyotype analysis was performed according to the method reported by Hu et al [22].

### Western Blot Analysis

Total protein was isolated from purified YMECs, fibroblast cells (negative control), and lactating mammary tissues (positive control) using a Q proteome mammalian protein isolation kit (Qiagen, USA). The lysates were separated in 12% polyacrylamide gels, and transferred onto a PVDF membrane. After blocking in TBS (20 mM Tris and 137 mM NaCl) containing 5% BSA, the membrane was incubated overnight at 4°C with rabbit anti-bovine casein antibody (Jingmei, China). The membrane was washed three times for 15 min each with TBST, and incubated with HRP-conjugated mouse anti-rabbit IgG (Beyotime, China) for 1 h at 37°C.

### Transfection of the EGFP Gene into YMECs

Cells were seeded in 12-well flat-bottom culture plates and cultured in antibiotic-free DMEM/F12 overnight. Linearized pEGFP-N1 (Clontech, USA) plasmid was transfected into YMECs using Lipofectamine 2000 (Invitrogen) for 4 h according to the manufacturer's instructions. The mixture was removed, and the cells were cultured with fresh DMEM-F12 until 90% confluence. Moreover, the transfected cells were selected with 500 µg/mL G418 (Sigma) in growth medium. Transfected monoclonal cells were obtained after one week.

### Infection Assay of *Staphylococcus aureus* to YMECs

*S.aureus* infection assay was performed using the *S. aureus* subsp. aureus (ATCC 27543) strain as reported by Téllez–Pérez et al [23]. In brief, YMEC monolayers (approximately 1×10^6^ cells in six-well dishes) were obtained, infected with *S.aureus* (MOI 100∶1), and incubated for 3 h at 37°C. To determine the effect of *S. aureus* on the transcription of antimicrobial peptides (i.e., TAP andBNBD5) and related apoptotic factors (i.e., BAX and BCL-2) after infection, we performed real-time PCR with SYBR Premix Ex Taq (Takara, Dalian, China) on a Bio-rad Connect detection system (Bio-rad, USA). The special primer sequences are described in Table 2. Reactions were performed in 25 µL of the reaction mixture consisting of 12.5 µL of 2×SYBR green II PCR master mix, 1.0 µL of each primer (10 µm), 1 µL of cDNA, and 9.5 µL of ddH_2_O. PCR was conducted at 95°C for 30 s, followed by 39 cycles of 95°C for 5 s, 58°C for 25 s, and 72°C for 25 s. The relative mRNA expression was calculated using the Delta–Delta Ct method [24], and fold changes were compared using one-way ANOVA and Newman–Keuls test.

**Table 2:** Relative-quantitative PCR primers sequences.

Gene	Primers	Melting temperature (°C)	Product size (bp)
TAP	F:5′- CTCCATCACCTGCTCCTCG-3′	58	160
	R:5′- GCCCAACACAGGTGCCAA-3′		
BNBD5	F:5′- GCCAGCATGAGGCTCCATC-3′	58.5	143
	R:5′- TTGCCAGGGCACGAGATCG-3′		
BAX	F:5′- TGGACATTGGACTTCCTTCG-3′	60	100
	R:5′- TGAGCACTCCAGCCACAAAG-3′		
BCL-2	F:5′- AGGGACGGGGTGAACTGG-3′	60	193
	R:5′- CATACAGCTCCACAAGG-3′		
GAPDH	F:5′- GC TGGTGCTGAGTAGTTGGTG-3′	60	220
	R:5′- TCTTCTGGGTGGCAGTGATGG-3′		

### Flow Cytometry

Apoptosis of *S. aureus*-infected YMECs was examined using AnnexinV–FITC/PI double staining assay. Non-infected and infected YMECs (approximately 10^5^ cells) were resuspended in 200 ∶L of AnnexinV–FITC binding buffer consisting of 5 µL of Annexin V–FITC and 5 µL of PI. The cells were then incubated at room temperature in the dark. Flow cytometry was performed within 1 h using a flow cytometer (Beckman, FC500, USA).

## Results

### Establishment and Growth Characteristics of YMECs

**Figure 1:**
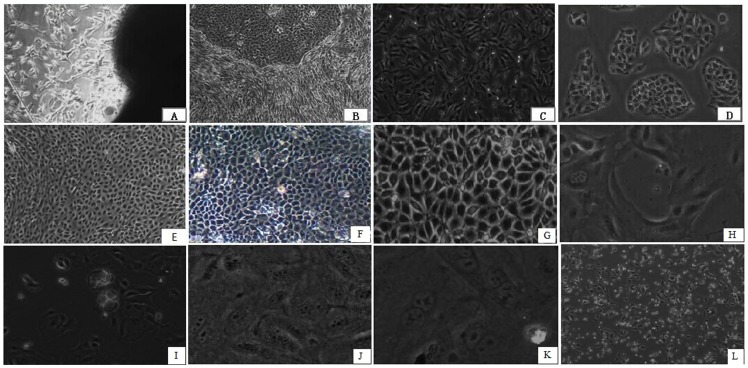
Morphology of YMECs. A: Fibroblasts elongated from tissues (40×). B: Contrast observation of YMECs and fibroblast cells cultured *in vitro* (40×). C: Purification of fibroblast cells (100×). D: YMECs formed islands when grown at low density (100×). E: Oval morphology of YMECs (100×). F: Typical cobble stone morphology of YMECs (100×). G: Irregular polygon morphology of YMECs (100×). H: Lumen-like structures of YMECs (200×). I: YMECs contained milk drops (200×). J: The cell nucleus was clearly observed in YMECs (400×). K: YMECs in mitotic phase (400×). L: Morphology of resuscitated YMECs (40×).

The entire growth process, including tissue culture and different culture stages, for isolating YMECs is shown in Fig. 1. Fusiform fibroblasts were first elongated from mammary cells after 5–6 d of culture (Fig. 1A). Primary epithelial cells were elongated from mammary tissues after 8–10 d in the culture, and surrounded by fibroblast cells (Fig. 1B). Selective trypsinization resulted in the removal of fibroblasts from epithelial cells. Subsequently, we obtained purified fibroblasts (Fig. 1C) and epithelial cells (Figs.1D–1G). YMECs formed islands when cultured at low density (Fig. 1D). Several morphologies, such as oval (Fig. 1E), typical cobblestone (Fig. 1F), and irregular polygon (Fig. 1G), emerged with cell growth. Moreover, a lumen-like structure formed among the cells (Fig. 1H), and some YMECs contained milk drops (Fig. 1I). The nucleus (Fig. 1J) and cells in the mitotic phase (Fig. 1K) were observed during cell culture. The cells obtained after freezing and thawing maintained normal morphology and growth characteristics (Fig. 1L).

### Growth Characteristics of YMECs

**Figure 2:**
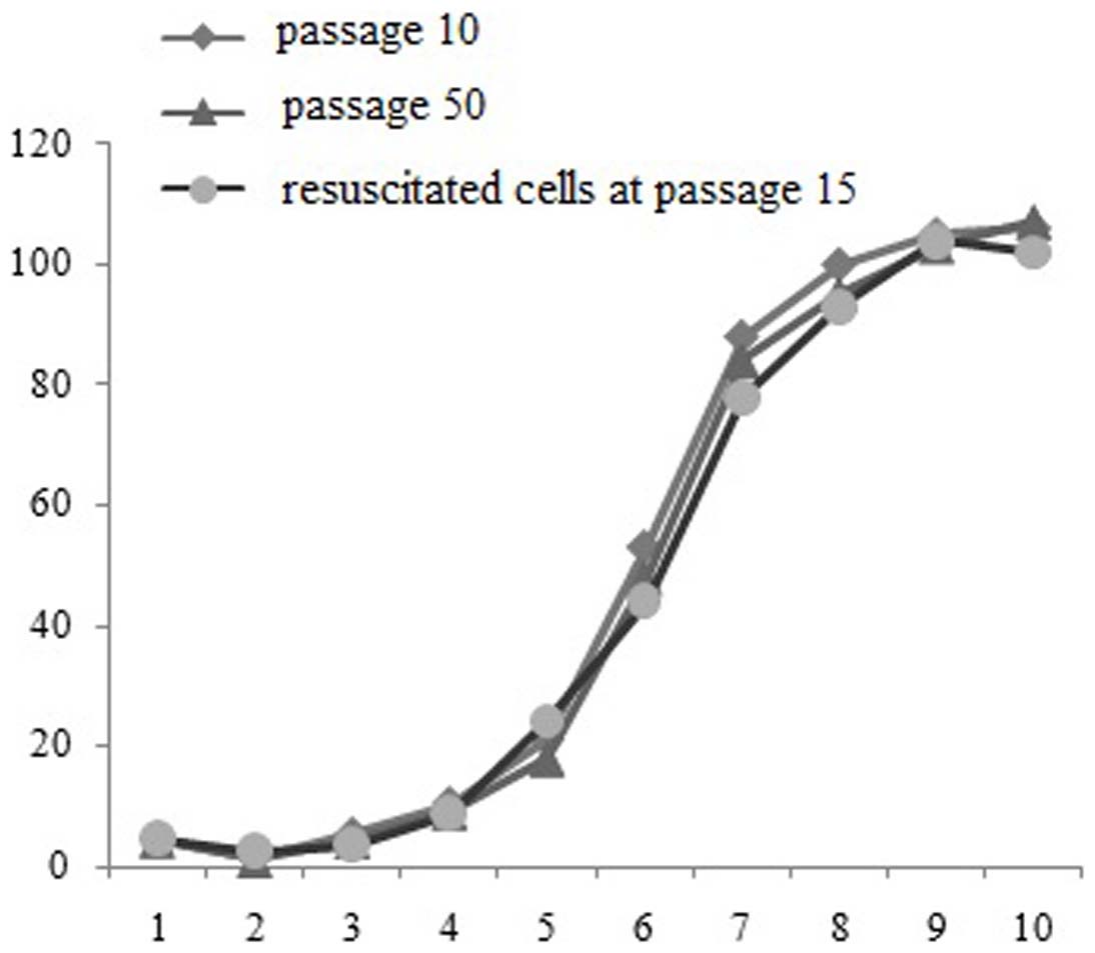
Growth curves of YMECs at the 10th, 60th, and 15th passages (resuscitated cells).

**Figure 3:**
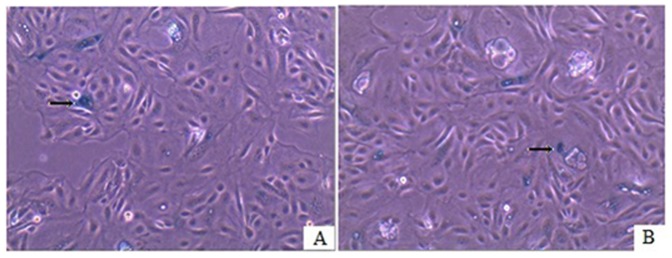
Analysis of cell senescence-associated β-galactosidase (SA-β-gal) activity in YMECs. A: SA-β-gal staining in early passage 10 YMECs (100×); B: SA-β-gal staining in late passage 60 (100×). Blue staining indicate the presence of senescence associated β-galactosidase activity (arrow).

Growth curve analysis of YMECs revealed a population doubling time of approximately 36–48 h without any substantial changes among the 10th (early), 50th (late), and 15th passages (frozen thawed MECs) (Fig. 2). The cultured YMECs slowly grew within the first 4 d, and rapidly proliferated from day 5 to day 8. After day 9, the proliferation of the cells became slow. The cell growth curve exhibited an “S” shape (Fig. 2). Cell contact inhibition was observed at post-confluence stage, during which many floating dead cells appeared. Survival rate analysis showed that cell adherence and the survival rate gradually increased within 24 h, and the cell survival rate reached 85% at 24 h. These findings indicate that YMECs exhibited high viability. Bacterial and mycoplasma analyses demonstrated that the isolated cells were not contaminated by bacteria or mycoplasma. Senescence Associated β-galactosidase (SA-β- gal) assay was carried on early passage (passage 10) and late passage (passage 60) YMECs to assess the level of cell senescence. SA-β- gal staining was observed in both early and late passage YMECs cells of which positive cells stained blue, moreover, approximately 10% of YMECs cells stained for SA-β- gal (Fig. 3).

### Cytoskeleton and Vimentin Protein Expression

**Figure 4:**
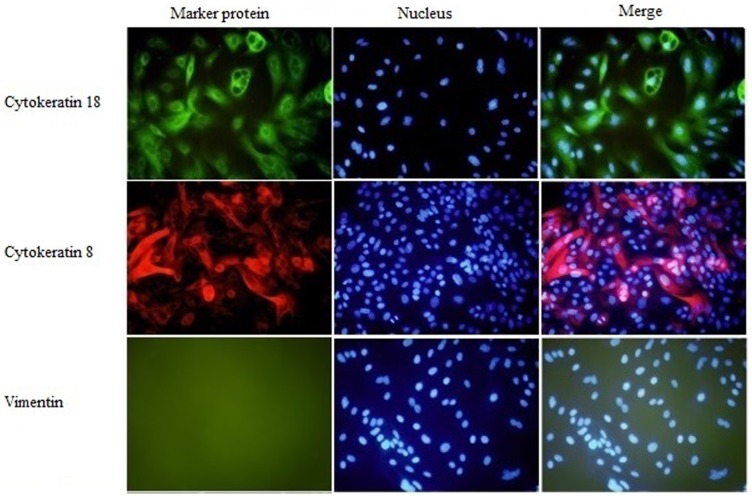
Immunofluorescence identification of the isolated YMECs. YMECs exhibited positive immunofluorescence results for cytokeratin8 andcytokeratin18, but negative results for vimentin.

Cell homogeneity was investigated by examining the protein expression of cytoskeletons 8 and 18, which are specific for epithelial cells, and vimentin, which is specific for stromal cells (such as fibroblast cell). Cells exhibited strong positive staining for cytokeratins 8 and 18 rather than vimentin, indicating that the cultured cells possessed the properties of epithelial cells (Fig. 4).

### Milk Protein Expression

**Figure 5:**
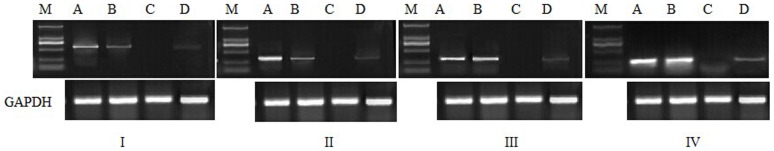
RT-PCR analysis for CSN2 (I), BT1N1A1(II), LTF(III), and BLG (IV) in YMECs. M: D2000 marker; A: yak mammary tissues (positive control); B: YMECs cultured in induction medium; C: fibroblasts (negative control); and D: YMECs cultured in basal medium.

**Figure 6:**
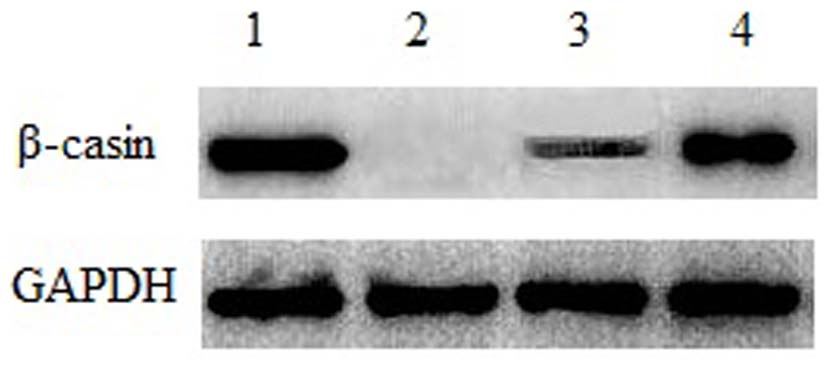
Western blot analysis of CSN2 in YMECS. 1: Yak mammary tissues (positive control); 2:fibroblasts (negative control); 3:YMECs cultured in basal medium; and 4:YMECs cultured in induction medium.

The expression of milk proteins in YMECs should be identified to determine whether they can mimic an in vivo system. We evaluated the protein synthesis of the isolated cells based on the mRNA and protein expression levels of milk protein. Total RNA and protein were extracted from mammary tissues, epithelial cells cultured in basal medium, epithelial cells cultured in induction medium, and fibroblast cells. RT-PCR was used to determine the mRNA expression of milk proteins (Fig. 5), whereas western blot analysis was applied to detect CSN2 protein expression (Fig. 6). RT-PCR and western blot indicated that the isolated YMECs could synthesize mammary-specific milk protein. Moreover, inducible factors in the medium could induce YMECs to synthesize milk protein *in vitro*.

### Chromosomal Analysis of YMECs

**Figure 7:**
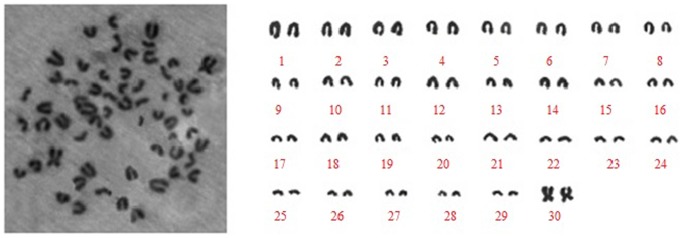
Chromosomal analysis of YMECs. Representative metaphase distributionand karyotype of YMECs showing 30 pairs of chromosomes specific to Yak (2*n* = 60).

Chromosomal analysis demonstrated that the isolated epithelial cells, including primary, purified, and resuscitated cells, exhibited normal diploid configuration with 60 chromosomes (Fig. 7).

### EGFP Gene Expression

**Figure 8:**
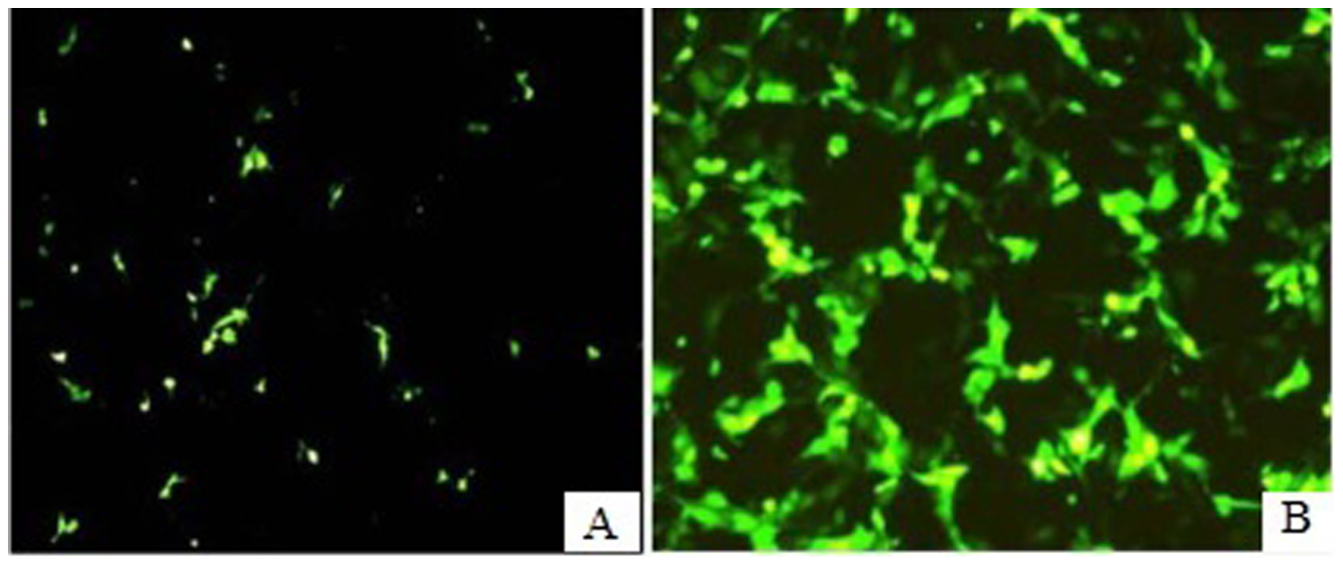
YMECs transfected with the EGFP gene. A: Transfected cells 24 h after transfection (100×); B: pure population of transfected YMECs after G418 selection (100×).

We successfully established YMECs transfected with the EGFP gene (Fig. 8A). A pure population of transfected cell line was obtained after G418 screening (Fig. 8B). This cell line could still express EGFP after the cells were subjected to 30 passages.

### Invasion Assay of *S.aureus*

**Figure 9:**
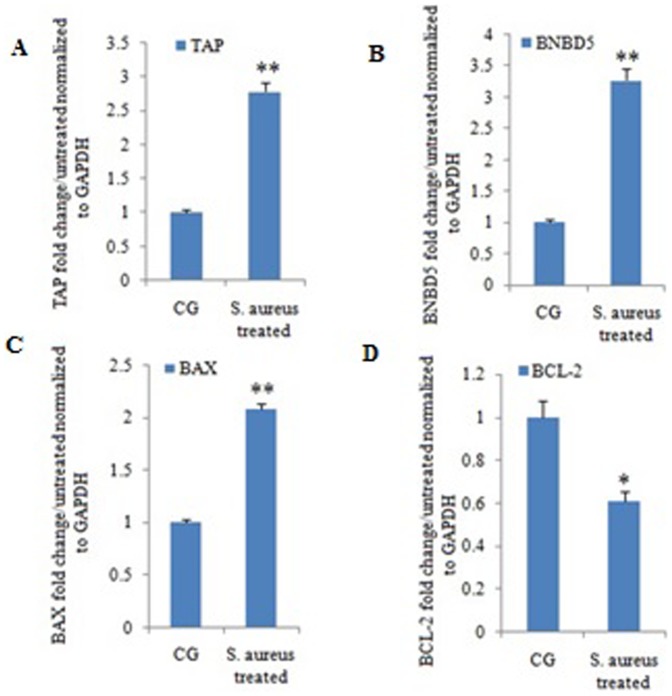
Real-time PCR analysis of antimicrobial peptides and apoptotic factors. CG: Control group without treatment; and *S.aureus* treated: cells were invaded by *S.aureus*. Each bar shows the mean ± SE of three independent experiments. GAPDH was selected as the internal control gene. The symbol “*” indicates significant variation (p<0.05) compared with the control cells, and “**” indicates highly significant variation (p<0.01).

**Figure 10:**
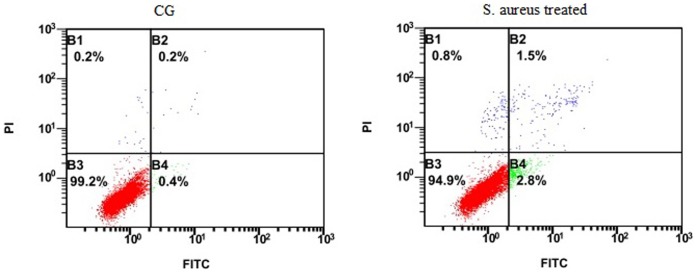
Flow cytometric analysis on *S.aureus*-induced apoptosis. CG: Control group without treatment; and *S.aureus* treated: cells were invaded by *S.aureus*.

To investigate the reaction of YMECs to *S. aureus* invasion, we performed real-time PCR to detect the expression levels of antimicrobial peptides (i.e., TAP and BNBD5) and apoptotic factors (i.e., BAX and BCL-2). The expression of antimicrobial peptide gene was induced when cells were challenged with *S. aureus* (Figs. 9A and 9B). Moreover, the expression of the apoptotic protein BAX significantly increased (Fig. 9C), whereas that of the anti-apoptotic protein BCL-2 decreased compared with those of the control group (Fig. 9D). Flow cytometry assay (AnnexinV–FITC/PI) was performed to determine whether apoptosis occurs in *S. aureus* infection-induced YMECs (Fig. 10). For infected YMECs, early apoptotic cells (annexin V^+^PI^−^) and post-apoptotic necrosis (annexin V^+^PI^+^) significantly increased compared with those of the control group (p<0.05).

## Discussion

This study aimed to establish a primary YMEC line and characterize its functional properties. This study is important because of its possible contributions to the study of yak mammary biology. The explant culture yielded a heterogeneous population of cells containing epithelial and fibroblast-like cells. Fibroblasts were removed from epithelial cells by digesting with trypsin according to their different sensitivities to trypsin. YMECs exhibited cobblestone morphology similar to the MECs isolated from other species, and formed islands when seeded at low density. Many MEC lines can form a dome-like structure when cultured on plastic substratum. Zheng et al. (2010) reported that pig MECs can form a dome-like structure on plastic containers [12]. The MECs isolated from cows can also form a dome-like structure on plastic containers [5]. However, the cow MAC-T cell line cannot form a dome-like structure when cultured on plastic containers, but exhibits the structure when cultured on collagen implements or cultured together with cow muscle epithelial cells [25]. The present study demonstrated that YMECs could forma dome-like structure. The formation of a dome-like structure in MECs suggested that the cells underwent contact differentiation and secreted basement membrane components. Remarkably, interconnecting structures with branching patterns were observed between domes in buffalo MECs (BuMECs), indicating that the contact-mediated differentiation of BuMECs extends from one dome to another [13].

Immortalisation is a process where cultured cells escape senescence and acquire the ability to grow in culture indefinitely [26]. Bovine and buffalo MECs have been reported to spontaneously overcome the proliferation barriers and gained immortalization [13], [25]. To examine the extent of senescence in YMECs, we performed SA-β-gal staining at passage 10 and 60. Results showed very few senescent cells existed among populated viable YMECs, implying the cells have undergone random transformation events and gained possible immortalization.

The epithelial morphology of the established YMECs was investigated. However, the specific expression of cytoskeleton proteins for epithelial lineage should be investigated by immunocytochemistry to further identify the cell line. Cytokeratins are intermediate filaments of epithelial cells and important protein markers to identify epithelial cells [27]. As a member of the intermediate filament gene family, cytokeratin 18, which generally exists with cytokeratin 8, is used to determine epithelial cell property [28]–[30]. The purified YMECs showed positive staining to cytokeratins 8 and 18, thereby providing direct evidence of their epithelial nature. Moreover, we further examined myoepithelial cell contamination using immunocytochemistry for vimentin, which is a protein marker of myoepithelial cells. The immunocytochemistry results confirmed the absence of myoepithelial cells in YMECs.

To ascertain the effect of frozen preservation on the isolated YEMCs, we compared the growth characteristics among the unpreserved early (10th passage), late (50th passage), and frozen thawed cells of YMECs on plastic substratum. Growth curves revealed no significant difference in the proliferation between unpreserved and frozen thawed cells. These established growth curves showed a typical “S” sigmoid with lag, exponential, and steady phases. The results suggest that frozen preservation did not affect the proliferation of the isolated YMECs. Similar observations have been reported in buffalo [13], bovine [22], and caprine [4].

Milk protein secretion is an important mammary-specific feature. Generally, insulin, hydrocortisone, and prolactin are used in culture media to induce milk protein expression [31]–[33]. In the present study, variations in marker gene expression between cells cultured in basal and induction media were observed, indicating that the difference in the composition of culture media may influence functional gene expression. The expression levels of the CSN2, BTN1A1, and LTF genes were determined using RT-PCR, whereas the presence of the CSN2 protein was examined by western blot analysis. These findings suggest that YMECs were functionally differentiated and exhibited normal secretory function. Anand et al. also determined the expression of the CSN2, CSN3, BTN1A1, and LTF genes in BuMECs cultured on plastic substratum. In experiments using bovine MEC cultures seeded on a plastic surface, the gene expression levels of CSN2 and BTN1A1 were also detected. YMECs showed properties similar to BuMECs [13] and bovine MECs [34] cultured on plastic substratum, and remained sensitive to lactogenic hormones. These results differed from the investigations of other researchers who reported that collagen is essential for milk protein secretion in MECs [35], [36]. Moreover, the transcript levels of CSN2, BTN1A1, and LTF did not significantly vary between mammary gland tissues and epithelial cells cultured in induction media; however, the transcript level in cells cultured in basal medium was evidently lower. These results suggest that these bioactive factors, including hormone in induction media, could upregulate the expression of milk proteins. Although many factors influencing gene expression were identified, the special functional mechanism remains unclear. Future studies should investigate the functional mechanisms of bioactive factors, and compare mammary cell culture models in different culture conditions.

Cow and goat mammary glands are usually selected as appropriate bioreactors for recombinant protein expression because of their high milk production [37]–[39]. To obtain transgenic animals, the main barrier is the modest expression of the recombinant gene. An *in vitro* screening system for examining superior transgenes prior to transfer is important to improve transgenic animal production. Thus, using fully functional and transfectable YMECs is preferable for rapid screening. In the present study, we investigated EGFP gene expression in YMECs, which could elucidate foreign gene integration and expression in positive transgenic cells for further studies on transgenic yak.

Bovine mastitis is one of the most common and costly diseases of dairy cattle worldwide. Among the broad spectrum of bacterial and fungal pathogens, *S.aureus* is one of the primary contagious pathogens responsible for mastitis [40]–[42]. The present study revealed that *S.aureus* infection could induce YMECs to express antimicrobial peptide for inhibiting bacterial growth, which was consistent with the results of previous work. BAX and BCL-2 function as death agonists and antagonists, respectively; their relative proportion controls the sensitivity or resistance of cells to apoptotic stimuli [43], [44]. Our results also demonstrate that BAX mRNA expression increased in the infected group compared with that in the control group. However, BCL-2 mRNA expression significantly decreased in the infected group. The relative proportion (BAX/BCL-2) was higher in the infected group, indicating that *S.aureus* could induce apoptosis in mammary glands. YMECs exhibited several cellular and biochemical hallmarks of apoptotic cells, including karyopyknosis, condensation, swelling, and development of apoptotic bodies. Annexin V–FITC/PI assay demonstrated that the apoptosis rates in the infected groups were higher than those of the control group. Therefore, the established YMECs are useful in future research on *S.aureus* invasion.

## Conclusion

A stable YMEC line that retained typical MEC properties was established, which may be useful as a *in vitro* model for studies on yak mammary gland development and differentiation. We then successfully established YMECs transfected with the EGFP gene, indicating that the cells could be used as an *in vitro* screening system for identifying superior transgenes prior to transfer. Bacterial infection assay showed that YMECs could be a potential tool for future research on the relationship between MECs and *S. aureus*.
